# Anti-tumor innate immunity activated by intermittent metronomic cyclophosphamide treatment of 9L brain tumor xenografts is preserved by anti-angiogenic drugs that spare VEGF receptor 2

**DOI:** 10.1186/1476-4598-13-158

**Published:** 2014-06-26

**Authors:** Joshua C Doloff, Chong-Sheng Chen, David J Waxman

**Affiliations:** 1 Division of Cell and Molecular Biology, Department of Biology, Boston University, 5 Cummington Street, Boston, MA 02215, USA

**Keywords:** VEGFR2, Metronomic chemotherapy, Innate immunity, DC101, Sorafenib

## Abstract

**Background:**

Metronomic cyclophosphamide given on an intermittent, 6-day repeating schedule, but not on an exposure dose-equivalent daily schedule, activates an anti-tumor innate immune response that leads to major regression of large implanted gliomas, without anti-angiogenesis.

**Methods and approach:**

Mice bearing implanted 9L gliomas were used to investigate the effects of this 6-day repeating, immunogenic cyclophosphamide schedule on myeloid-derived suppressor cells, which are pro-angiogenic and can inhibit anti-tumor immunity, and to elucidate the mechanism whereby the innate immune cell-dependent tumor regression response to metronomic cyclophosphamide treatment is blocked by several anti-angiogenic receptor tyrosine kinase inhibitors.

**Results:**

Intermittent metronomic cyclophosphamide scheduling strongly increased glioma-associated CD11b^+^ immune cells but not CD11b^+^Gr1^+^ myeloid-derived suppressor cells, while bone marrow and spleen reservoirs of the suppressor cells were decreased. The inhibition of immune cell recruitment and tumor regression by anti-angiogenic receptor tyrosine kinase inhibitors, previously observed in several brain tumor models, was recapitulated in the 9L tumor model with the VEGFR2-specific inhibitory monoclonal antibody DC101 (p < 0.01), implicating VEGFR2 signaling as an essential step in metronomic cyclophosphamide-stimulated immune cell recruitment. In contrast, sorafenib, a multi-receptor tyrosine kinase inhibitor with comparatively weak VEGF receptor phosphorylation inhibitory activity, was strongly anti-angiogenic but did not block metronomic cyclophosphamide-induced innate immunity or tumor regression (p > 0.05).

**Conclusions:**

The interference by receptor tyrosine kinase inhibitors in the immunogenic actions of intermittent metronomic chemotherapy is not a consequence of anti-angiogenesis *per se*, as demonstrated in an implanted 9L tumor model. Furthermore, this undesirable interaction with tyrosine kinase inhibitors can be avoided by using anti-angiogenic drugs that spare the VEGFR2 pathway.

## Background

Clinical strategies that use cytotoxic drugs to target tumor cells for destruction are frequently confounded by the responses of tumor-associated stromal cells, which can lead to stimulation of tumor growth, angiogenesis, invasion and metastasis and an immune suppressive environment [1]. Cancer chemotherapy delivered on a metronomic schedule offers a novel approach to this problem by combining direct tumor cell cytotoxicity with repeated disruption of the tumor microenvironment. Clinical metronomic protocols primarily utilize daily low dose schedules and most commonly employ the classic cytotoxic drug cyclophosphamide (CPA) [2,3]. Metronomic administration of CPA and other cancer chemotherapeutic agents is thought to improve anti-tumor activity by combining direct tumor cell drug toxicity with tumor endothelial cell-directed anti-angiogenesis while minimizing toxicity to the patient [4-7]. Recent findings implicate additional mechanisms in the action of metronomic chemotherapy, most notably immune-based mechanisms [2,8], including the activation of innate immunity by intermittent metronomic drug scheduling [9,10]. Thus, CPA administered on an intermittent, every 6-day metronomic schedule stimulates tumor recruitment of macrophages, natural killer (NK) cells, and dendritic cells with regression of large established tumors, as seen in several implanted glioma models [10]. This potent innate anti-tumor immune response is not achieved using a traditional maximum tolerated dose schedule [10], nor is it seen using an AUC-equivalent daily low dose metronomic CPA schedule [9] that models daily metronomic schedules commonly used in the clinic [2,3].

Several cancer chemotherapeutic drugs have the potential to stimulate immunogenic cell death, independent of metronomic scheduling [11,12]. CPA promotes bone marrow generation of dendritic cell precursors capable of antigen presentation and differentiation of T-helper 17 cells [13,14], while doxorubicin can induce CD8 T-cell activation and interferon-γ production [15]. Moreover, CPA, paclitaxel, temozolomide and vinorelbine can deplete regulatory T-suppressor cells [16-19] and metronomic schedules of docetaxel and gemcitabine suppress myeloid-derived suppressor cells (MDSCs) [20,21]. MDSCs inhibit NK cell activity [22] and thus have the potential to counter the NK cell-dependent regression of tumors treated with CPA on an intermittent metronomic schedule [10]. Here, we investigate the effects of intermittent metronomic CPA treatment on tumor-associated MDSCs and on MDSC reservoirs in bone marrow and spleen.

Prior studies found that anti-angiogenesis drugs with VEGF receptor tyrosine kinase inhibitory activity block metronomic CPA-activated anti-tumor innate immunity and the associated tumor regression response [10]. This inhibition of immune cell recruitment could result from the loss of tumor blood vessels trafficking immune cells into the tumor compartment. Alternatively, it could be a more direct consequence of the inhibition of VEGF signaling, which is common to both endothelial and immune cell lineages [23] and is important for dendritic cell-endothelial cell cross-talk, trans-differentiation [24] and tumor-associated macrophage infiltration [25]. Endothelial cell VEGF signaling is also important for chemokine expression and secretion in pro-inflammatory responses [26], which may be important for metronomic CPA-stimulated anti-tumor immunity [9,10]. A third possibility, suggested by the off-target effects of many receptor tyrosine kinase inhibitors (RTKIs), is that receptors other than VEGF receptor (VEGFR), such as C-FMS/CSF1-R on macrophages [27] and FLT3 on dendritic cells and NK cells [28], are involved in the observed innate immune cell inhibition. We presently investigate these issues using the VEGFR2-specific inhibitory monoclonal antibody DC101 [29], which blocks VEGFR2-dependent angiogenesis without off-target effects.

The inhibition of intermittent metronomic CPA-activated anti-tumor innate immunity by VEGF receptor-targeted anti-angiogenic drugs [10] indicates a need for therapies that circumvent this inhibition. One approach is to employ anti-angiogenesis drugs that act through mechanisms independent of VEGF receptor. Presently, we consider sorafenib [30], a multi-RTKI with an IC_50_ for VEGFR2 > 100-fold higher than the IC_50_ values of the VEGFR-selective RTKIs axitinib (AG-013763), cediranib (AZD2171), and AG-028682 [31-34], all of which strongly inhibit metronomic CPA-induced anti-tumor immunity and tumor regression [10]. Our findings show that sorafenib is highly anti-angiogenic, yet it does not interfere with tumor recruitment of innate immune cells or metronomic CPA-induced tumor regression, supporting the conclusion that inhibition of innate immune cell recruitment is not an intrinsic feature of tumor anti-angiogenesis.

## Results

### Metronomic CPA depletes MDSCs from bone marrow and spleen

**Figure 1:**
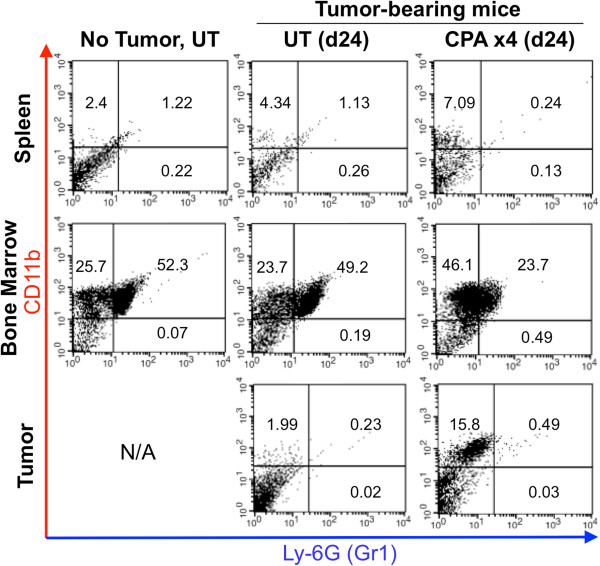
**FACS analysis of CD11b**^**+ **^**cells and Gr1**^**+**^**CD11b**^**+ **^**MDSCs.** Ly-6G (Gr1)^+^, CD11b^+^, and Gr1^+^CD11b^+^ co-positive cells were analyzed in single-cell suspensions prepared from untreated (UT) and metronomic CPA-treated (CPA) spleens, bone marrow and 9L tumors from *scid* mice euthanized 6 days after the 4^th^ CPA cycle (day 24). Cell numbers in each quadrant are expressed as a percentage of the total cell population. Metronomic CPA significantly increased single CD11b-positive populations in spleen and bone marrow (p < 0.05) and tumor (p < 0.001), but decreased Gr1-CD11b co-positive populations in bone marrow (by 2-fold; p < 0.05) and spleen (by 4.7-fold; p < 0.001) (n = 2 per treatment group), with no significant increase in treated tumors (n = 4). IgG background for Gr1 (spleen: 0.06%, bone marrow: 0%, and tumor: 0.01%), CD11b (spleen: 0.22%, bone marrow: 0.11%, and tumor: 0.34%), and Gr1-CD11b co-positive (spleen: 0.06%, bone marrow: 0.02%, and tumor: 0.02%). Also see Additional file 1. Each treatment group was repeated at least 2–3 times.

MDSCs are increased in tumor-bearing mice, and in cancer patients, and have been implicated in promoting tumor growth and suppressing anti-tumor immunity [35]. Given the ability of MDSCs to suppress NK cell activity [22], which contributes functionally to metronomic CPA-induced tumor regression [10], we investigated whether MDSCs are also recruited into CPA-treated tumors, where they could counter the innate immune response to metronomic chemotherapy. FACS analysis of MDSCs was performed on single-cell suspensions prepared from untreated and metronomic CPA-treated spleens, bone marrow and 9L tumor xenografts grown in *scid* mice. CD11b^+^ was used as a marker of bone marrow-derived cells, including monocytes, macrophages, dendritic cells and NK cells, while CD11b^+^Gr1^+^ co-positive cells marked MDSC populations [36]. The presence of 9L tumors had no effect on the distribution of either single-positive CD11b^+^ cells or double-positive CD11b^+^Gr1^+^ cells in either spleen or bone marrow (Figure 1, *left* vs. *middle* column). Single-positive CD11b^+^(Gr1^−^) cells were increased significantly – by ~2-fold in spleen and bone marrow and by ~8-fold in tumor after 4 cycles of CPA treatment (day 24) (Figure 1, *middle* vs. *right* column, *top left* quadrant). A time-dependent increase in CD11b^+^ tumor-infiltrating cells was seen from 2 to 4 CPA cycles (Additional file 1). Metronomic CPA significantly decreased CD11b^+^Gr1^+^ MDSC populations in treated bone marrow (2-fold decrease) and in treated spleens (4.7-fold decrease), with no significant increase in the treated tumors (Figure 1, *middle* vs. *right* column: *top right* quadrant). Thus, metronomic CPA suppresses CD11b^+^Gr1^+^ MDSC populations in spleen and bone marrow without significantly increasing the intratumoral MDSC population.

### VEGFR2-specific inhibitor DC101 blocks metronomic CPA-induced tumor regression

**Figure 2:**
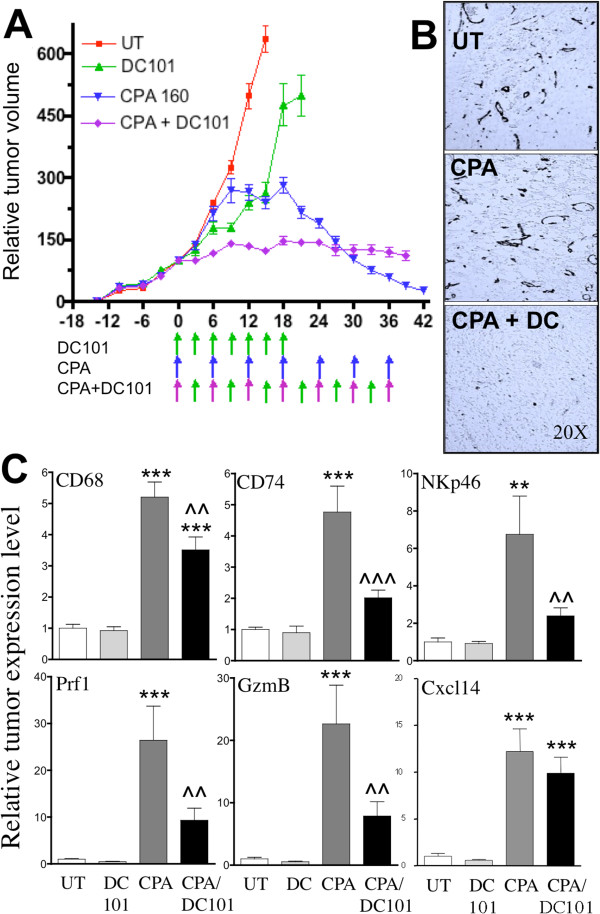
**DC101 inhibits metronomic CPA-induced 9L tumor regression and anti-tumor immunity. A)** 9L tumors were treated with metronomic CPA (160 mg/kg i.p. every 6 days; bottom 2 sets of arrows along x-axis), DC101 (22.5 mg/kg BW, i.p. every 3 days up to day 18; top set of arrows), or DC101 in combination with CPA (bottom set of arrows). Shown are mean tumor volumes, mean ± SE for n = 10-12 individual tumors/treatment group. The DC101 + CPA combination group was terminated after 13 injections (day 39) due to toxicity, as indicated by body weight loss (data not shown). CPA vs. CPA + DC101: *p* < 0.01 by 1-way ANOVA comparing time points after 30 days. **B)** Immunostaining of endothelial cell marker CD31 in untreated 9L tumors (UT) or 9L tumors treated with metronomic CPA ± DC101 and isolated on treatment day 24. Representative images are shown at 20X magnification, with background color tone adjusted to light blue to highlight the CD31-immunostained blood vessels and their near total absence in the CPA + DC101 treated tumors. Signal intensities quantified by ImageJ indicate that DC101 decreases CD31 staining to 21% of control (*p* < 0.001) (data not shown). **C)** qPCR analysis of mouse marker genes CD68 (macrophages), CD74 (dendritic cells), NKp46 (NK cells), NK cell cytotoxic effectors perforin (Prf1) and granzyme B (GzmB), and NK cell chemoattractant Cxcl14 in 9L tumor xenografts treated (as in panel A) and extracted on treatment day 15 (untreated tumors, *UT*), and at a time point corresponding to 4 CPA cycles (DC101 day 21, CPA day 24, CPA + DC101 day 24). All qPCR data are mean ± SE values for n = 10-16 tumors/group. **, ***, *p* < 0.01 or 0.001, treatment versus UT; ^^, ^^^, *p* < 0.01 or 0.001, for CPA + DC101 versus CPA alone, by 1-way ANOVA. Results shown are representative of two independent studies.

**Figure 3:**
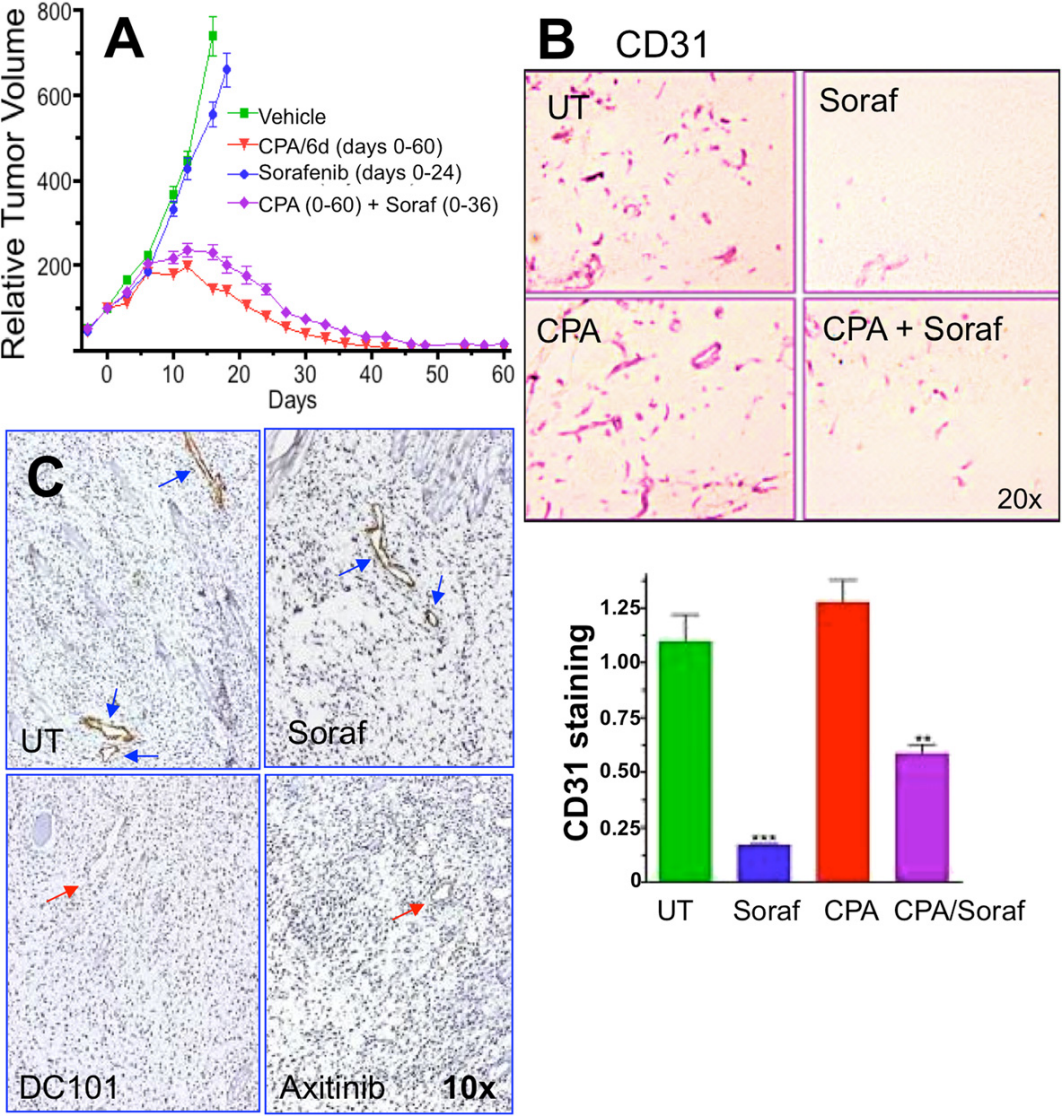
**Sorafenib inhibits angiogenesis without blocking metronomic CPA-induced tumor regression. A)** Mice bearing 9L tumors were drug-treated, as indicated, with tumor volumes normalized to 100% at the first day of treatment (day 0), when group averages reached ~500 mm^3^ (n = 12 tumors/group). Drug treatments: sorafenib (25 mg/kg, i.p. daily for 24 or 36 days, as marked, and metronomic CPA (140 mg/kg i.p. every 6 days for 11 cycles; days 0–60). Data shown are normalized tumor volumes, mean ± SE for n = 10-12 tumors/group. CPA vs. CPA + Soraf: *p* > 0.05 by 1-way ANOVA. **B)** Representative CD31 immunostaining images of tumors treated as in A and excised on treatment day 24. ImageJ quantification is shown below, mean ± SE based on n > 20 images/treatment group, with **, *p* < 0.01; ***, *p* < 0.001 vs. untreated (UT) controls by 1-way ANOVA. **C)** Phospho-VEGFR2 immunostaining (brown) with hematoxylin counterstaining (purple) of 9L tumors collected from mice either 2 or 4 hr after a single injection of DC101, sorafenib, or axitinib, or from untreated (UT) tumors (see Methods). Blue arrows, phospho-VEGFR blood vessels; red arrows, unstained blood vessels. Results are based on >20 images taken over two independent sections in each of 4 tumors per treatment group. Results shown are representative of at least two separate sets of analyses. See Additional file 4 for additional images.

Metronomic CPA treatment on an intermittent, 6-day repeating schedule regressed large, established 9L gliosarcoma xenografts in *scid* mice after 3–4 cycles of CPA administration (Figure 2A), in agreement with earlier findings [37]. Combination of metronomic CPA with the VEGFR2-specific monoclonal antibody DC101 (22.5 mg/kg) resulted in tumor stasis but little or no tumor regression over the 39-day observation period (Figure 2A). A very similar tumor growth static response was seen previously when metronomic CPA was combined with the VEGF receptor-selective inhibitor axitinib [38]. DC101 was a highly effective anti-angiogenic agent, as shown by the large decrease in CD31 immunostained blood vessels in the CPA and DC101 co-treated tumors (Figure 2B), but caused only a modest tumor growth delay, consistent with the relative insensitivity of 9L tumors to angiogenesis inhibition [38] (also see Figure 3A, below). DC101 significantly inhibited the CPA-stimulated tumor recruitment of macrophages (CD68 marker), dendritic cells (CD74 marker), and NK cells (NKp46 marker) and their cytotoxic effectors, perforin, granzymes, and lysozymes (Figure 2C; Additional file 2). These findings were confirmed by immunohistochemical staining for macrophages, NK cells, and the NK cytotoxic effector perforin 1 (Additional file 3). Metronomic CPA-induced expression of CXCL14, an NK cell chemoattractant, was not significantly affected by DC101 (Figure 2C). In a separate experiment where the DC101 dose was increased to 28.6 mg/kg, the inhibition of immune cell recruitment was even more complete but was accompanied by host toxicity in the CPA combination group (i.e., internal bleeding and death in 2 of 8 mice by treatment day 24; data not shown). Given the high specificity of DC101 for VEGFR2 [29], these studies demonstrate that VEGFR2 signaling contributes to metronomic CPA-induced anti-tumor innate immunity, and is likely the target in the previously observed inhibition of immune recruitment and tumor regression by three VEGF receptor-selective RTKIs [10].

### Sorafenib exerts anti-angiogenesis without blocking metronomic CPA-induced tumor regression

Sorafenib is a multi-RTKI with an IC_50_ for VEGFR2 > 100-fold higher than VEGF receptor-selective RTKIs (Table 1). When given at a dose of 25 mg/kg/day, sorafenib alone exhibited little or no activity against 9L xenografts when compared to vehicle-treated controls (Figure 3A), similar to DC101 (Figure 2A) and other anti-angiogenic drugs [38]. Importantly, when combined with metronomic CPA, sorafenib slightly delayed but did not block tumor regression (Figure 3A). Tumor regression was slightly less complete in the sorafenib + CPA combination group, but the overall anti-tumor response was not significantly different from that of metronomic CPA alone (p > 0.05, one-way ANOVA). This contrasts with the significant inhibitory effects of DC101 (Figure 2A) and the VEGFR-selective RTKIs axitinib, cediranib, and AG-028262 on metronomic CPA-induced tumor regression in the same tumor model [10,38]. The time of onset of tumor regression (~4-6 days after the third CPA treatment on day 12) was only slightly delayed (~3-6 day delay; range for individual tumors) by sorafenib co-treatment. The absence of a synergistic anti-tumor response to the sorafenib + metronomic CPA combination may reflect the low intrinsic sensitivity of 9L tumor growth to anti-angiogenesis (Figures 2A, 3A, and [38]) together with the strong tumor cytotoxic activity of CPA in conjunction with the potent anti-tumor immune response that it activates, which may already induce a maximal regression response. Both CPA alone and the CPA + sorafenib combination were well tolerated, with no significant toxicity apparent (data not shown).

**Table 1:** Specificities of receptor tyrosine kinase inhibitors

**IC**_**50**_**s**	**VEGFR1 (nM)**	**VEGFR2 (nM)**	**VEGFR3 (nM)**	**PDGFR (nM)**	**c-kit (nM)**	**Refs**
Axitinib	0.1	0.06-0.1 (0.25)	0.1-0.3	3-30	1-2	[31]
AG-028262	~0.1	<1 (0.34)	~0.1-0.3	193		[39,40]
Cediranib (AZD2171)	5	<1 (~0.5)	<1-3	5	2	[32]
Sorafenib	~26	15-90	~10-30	~10-30	68	[30,33]

Shown are IC_50_ values reported in the indicated references for inhibition of each human receptor protein. IC_50_ values for inhibition of mouse VEGFR2 are shown in parenthesis.

Sorafenib exhibited strong anti-angiogenic activity under these treatment conditions, as indicated by significant decreases in tumor microvessel density (CD31 immunostaining; Figure 3B). Tumor vascularity was also significantly decreased by sorafenib combined with metronomic CPA, albeit less completely than following sorafenib treatment alone (Figure 3B). This may reflect concomitant chemotherapy-induced circulating endothelial progenitor mobilization [41]. Metronomic CPA treatment alone did not decrease 9L tumor microvessel density (Figure 3B), as also seen earlier [9]. Immunostaining of 9L tumor samples with antibody to phospho-VEGFR2 (Tyr1214), a major site of VEGF-induced auto-phosphorylation, verified that sorafenib did not inhibit tumor blood vessel VEGFR2 phosphorylation (Figure 3C; Additional file 4). In contrast, DC101 and axitinib both inhibited VEGR2 phosphorylation when compared to the drug-free tumor controls (Figure 3C; Additional file 4). Thus, the anti-angiogenic activity of sorafenib under the *in vivo* treatment conditions used here likely involves one or more of its non-VEGFR targets.

### Sorafenib does not block metronomic CPA-induced anti-tumor innate immunity

**Figure 4:**
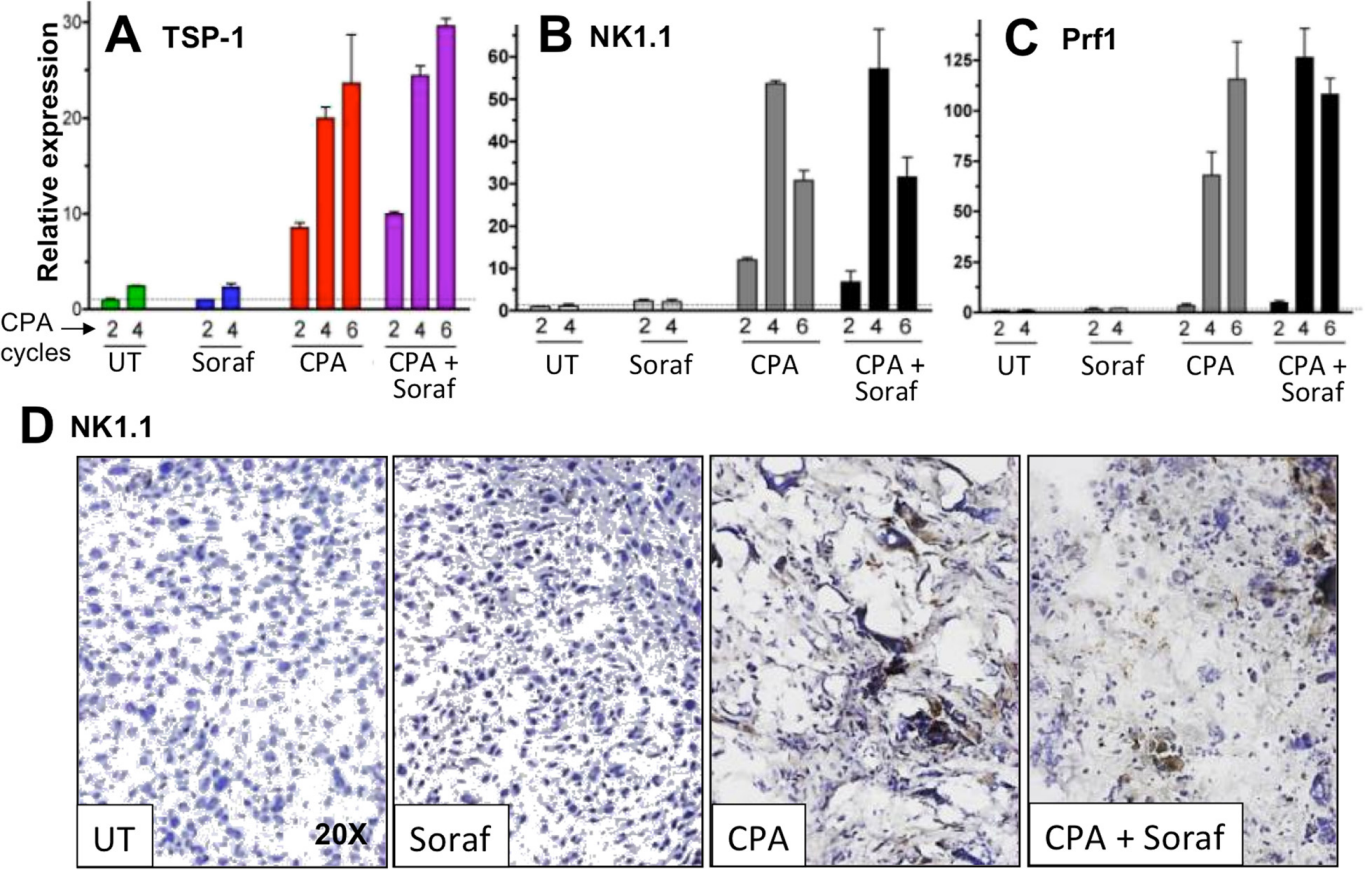
**Sorafenib does not block metronomic CPA-stimulated recruitment of innate immune cells to 9L tumors. (A, B, C)** qPCR analysis of mouse (host) TSP1, NK1.1 and perforin 1 in 9L tumors treated with vehicle, sorafenib alone, or metronomic CPA ± sorafenib as in Figure 3A and isolated 6 days after the 2^nd^, 4^th^, and 6^th^ CPA cycles (i.e., treatment days 12, 24, and 36), as indicated. Bars, mean ± SE for n = 5-6 tumors/group. Horizontal dashed line: expression level in untreated (UT) group on day 12. **(D)** Representative immunostained images of NK cell marker NK1.1, with hematoxylin counterstaining, in tumors collected on treatment day 24.

**Figure 5:**
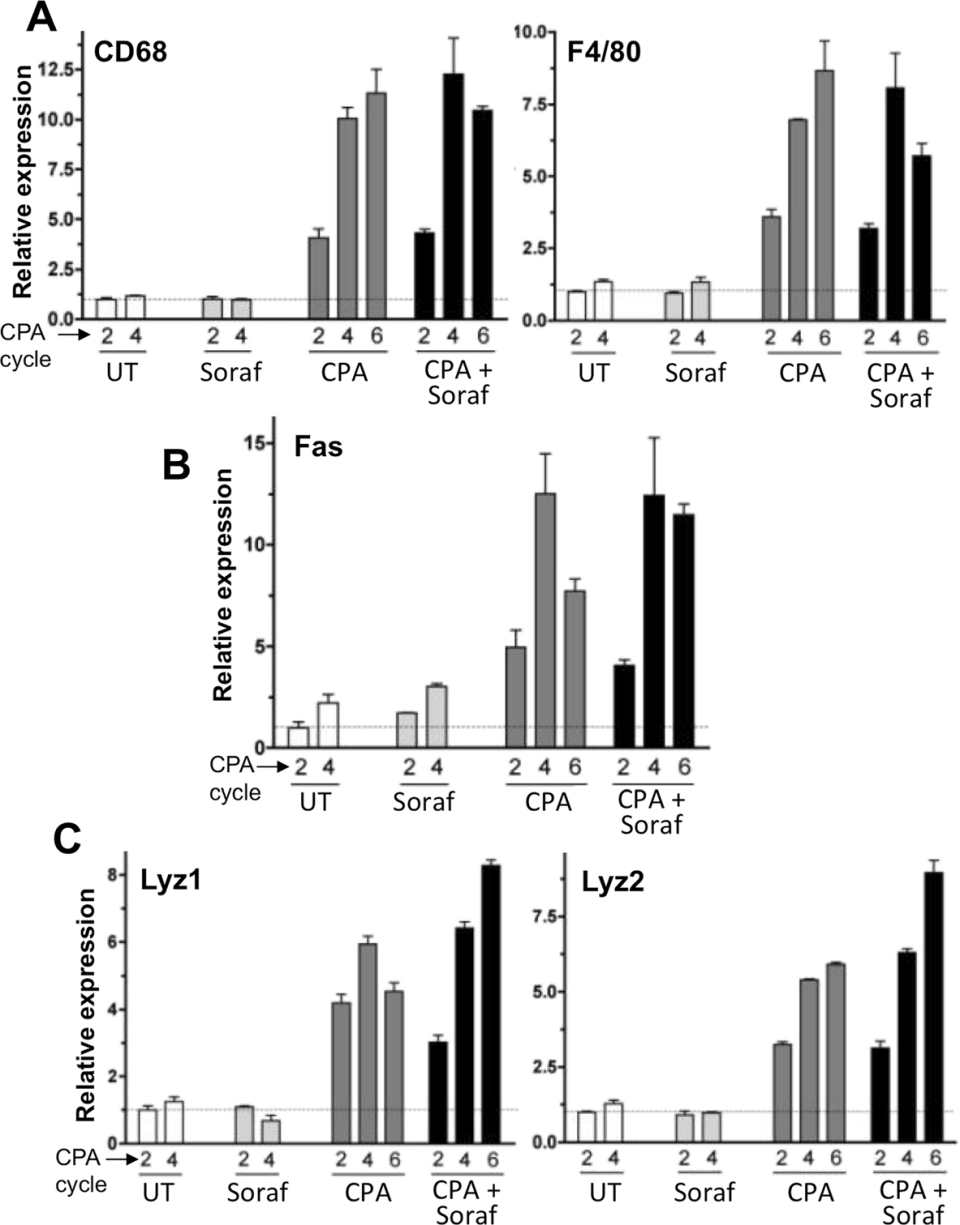
**Macrophage markers in metronomic CPA and sorafenib co-treated tumors.** qPCR analysis of 9L tumor RNA for host (mouse) macrophage markers CD68 and F4/80 **(A)**, death receptor Fas **(B)**, and cytotoxic effectors lysozymes 1 and 2 **(C)**. Samples were as described in Figure 4.

Next, we examined the effects of sorafenib on metronomic CPA induction of several factors associated with the innate immune response linked to tumor regression. Figure 4A shows that sorafenib does not block the strong increase in host (mouse) expression of thrombospondin-1 (TSP1), which occurred as early as 6 days after the second metronomic CPA treatment cycle. Sorafenib also did not interfere with NK cell recruitment, as determined by NK1.1 expression (Figure 4B) and NK1.1 immunostaining (Figure 4D). A similar time course of induction and lack of inhibition by sorafenib characterized perforin 1 (Figure 4C), an essential NK cell cytotoxic effector [42,43]. These findings contrast with the strong inhibition of metronomic CPA-induced NK cell recruitment by VEGF receptor-selective inhibitors, including VEGFR2-targeted DC101 (Figure 2C; Additional file 2, Additional file 3) and the pan-VEGFR inhibitory small molecules axitinib, cediranib and AG-028262 [10]. Furthermore, sorafenib did not inhibit tumor recruitment of macrophages (CD68 and F4/80 markers; Figure 5A), Fas, and lysozymes 1 and 2 (Figure 5B, 5C), which are important for macrophage anti-tumor activity [44,45]. Sorafenib also did not block the metronomic CPA-stimulated increases in dendritic cells (CD74), NK and dendritic cell hybrid interferon-producing killer dendritic cell factor B220 [46], platelet-associated platelet factor 4 (PF4), stromal-derived factor 1α (SDF1α), and CD11b, a general marker for bone marrow-derived cells (Additional file 5).

## Discussion

Metronomic CPA activates a strong anti-tumor innate immune response that leads to major tumor regression when given on an intermittent, 6-day repeating schedule, but not when using a dose-equivalent daily schedule [9], as seen in implanted glioma models [10]. The strong innate immune modulatory effects of this intermittent metronomic CPA regimen are not accompanied by anti-angiogenesis, are seen in both xenograft (rat 9L gliosarcoma and human U251 glioblastoma grown in scid mice) and syngeneic tumor models (mouse GL261 gliomas grown in fully immune competent C57BL/6 mice), and are inhibited by anti-angiogenic drugs that target VEGF receptor tyrosine kinases [10]. Here, we selected one of these tumor models, 9L, for further elaboration on the role of VEGFR2 signaling for anti-tumor innate immune function, based on its slower innate immune cell recruitment kinetics [10], which allowed for a longer time period to elucidate early events integral to this response.

While the anti-angiogenic small molecule RTKIs used in our earlier work [10] show selectivity for VEGF receptors, all RTKIs can be expected to have some off-target activity, suggesting that other receptors could be responsible for the observed inhibition of immune cell recruitment. Here, we report that the VEGFR2-specific inhibitory monoclonal antibody DC101 inhibits metronomic CPA-induced anti-tumor innate immunity and blocks tumor regression, implicating VEGFR2 in the anti-tumor innate immune cell response stimulated by intermittent metronomic CPA treatment. We also show that the interference of VEGF receptor inhibitors with anti-tumor innate immunity can be circumvented by using sorafenib, a multi-RTKI with greatly reduced VEGFR2 inhibitory activity compared to VEGF receptor-targeted anti-angiogenic drugs, which largely spares VEGFR from inhibition (Table 1) [32,34]. Thus, the undesirable antagonism previously seen between anti-angiogenic drugs and anti-tumor innate immunity activated by intermittent metronomic chemotherapy [10] can be avoided by using angiogenesis inhibitors that act *via* pathways independent of VEGFR2, which was found here to be essential for metronomic CPA-activated anti-tumor innate immunity.

Metronomic CPA increased tumor levels of CD11b, which marks bone marrow-derived innate immune cells, including monocytes/macrophages, dendritic cells and NK cells [47], but when co-expressed with a second marker, Gr1, identifies bone marrow MDSCs [35], which populate distant sites ahead of colonizing metastatic cells, creating conditions favorable for metastatic growth [48]. MDSCs have also been linked to tumor immune evasion [35], which may facilitate tumor metastasis. We found that metronomic CPA suppressed CD11b^+^/Gr1^+^ MDSC reservoirs in bone marrow and spleen, consistent with reports for metronomic schedules of other cancer chemotherapeutic drugs [20,21], but did not significantly increase tumor-associated MDSCs, which have the ability to counter innate immunity by suppressing NK cell activity [22].

Metronomic CPA treatment induced large increases in tumor-associated lysozymes 1 and 2, effectors of macrophage cytotoxicity, suggesting an additional mechanism of metronomic CPA-induced tumor cell lysis that is distinct from the NK cell perforin-mediated granzyme lysis mechanism described earlier [10]. Macrophages release many cytolytic factors, including lysozymes, following treatment with cancer chemotherapeutic drugs, leading to increased tumoricidal activity through filopodia extension and contact formation with target tumor cells [49]. We also observed metronomic CPA-stimulated increases in expression of B220, a marker for interferon-producing killer dendritic cells and NK-dendritic cells [46], suggesting these hybrid innate-adaptive immune cells contribute to innate immune cell-based tumor regression. Interferon-producing killer dendritic cells can kill cancer cells in their own right, but like dendritic cells, they also have the ability to present antigens, bridging innate immune killing and activating adaptive T- and B-cell responses [46].

The present studies were designed to address several questions raised by our prior finding that VEGF-directed RTKIs can block metro-CPA-induced innate immune responses [10], namely, whether the observed immune inhibition was: (a) due to the loss of tumor vascularity and hence a route to traffic immune cells into the tumor; (b) a result of off-target effects of the RTKIs (i.e., inhibition of kinases other than VEGFRs); or (c) a consequence of VEGFR inhibition unrelated to anti-angiogenesis. We have now addressed these issues by showing: (a) sorafenib induces a major loss of tumor vascularity without inhibiting innate immune cell recruitment, demonstrating that the loss of vascularity *per se* is not the cause of the innate immune cell inhibition; and (b) monoclonal antibody DC101 – which is highly specific for VEGFR2 – recapitulates the immune response inhibitory effects of the small molecule RTKIs that we reported earlier, indicating that innate immune cell inhibition is not the consequence of RTKI off-target effects. Further, (c) our studies with DC101 provide strong evidence that VEGFR2 inhibition, unrelated to the loss of vasculature, is the cause of the observed immune inhibition, establishing VEGFR2 as an essential factor in the innate immune cell response.

Although sorafenib is reported to have VEGFR inhibitory activity based on *in vitro* analysis and studies with cultured cell lines [30,33], that inhibition occurs at IC_50_ values >100-fold higher than inhibition by the three VEGF receptor-selective inhibitors examined previously [10] (Table 1). Indeed, we found that sorafenib did not inhibit VEGFR tyrosine phosphorylation in tumor blood vessels *in vivo* at the dose used in the present study (Figure 3C). In fact, very few studies have reported sorafenib inhibition of VEGFR phosphorylation in either implanted tumor models or in cancer patients *in vivo*, and even in those cases where inhibition was reported, the observed decreases in phospho-VEGF were partial, were not localized to tumor blood vessels, and mirrored the general decreases in tumor blood vessel density that result from sorafenib treatment [50-52]. Nevertheless, sorafenib was highly anti-angiogenic in our experiments, presumably due to inhibition of its other tyrosine kinase targets important for tumor angiogenesis, including Raf, c-Kit, PDGFR-β and Flt-3 [33]. Thus, sorafenib may be used under conditions where VEGFR2 is spared to avoid inhibition of the VEGFR2-dependent anti-tumor innate immune responses stimulated by intermittent metronomic CPA treatment.

Other anti-angiogenesis drugs that operate by a VEGFR2-independent mechanism may similarly be combined with intermittent metronomic therapies. These include Oxi4053, an endothelial cell tubulin-targeting cytotoxic agent, and TPN-470, which induces endothelial cell cell-cycle arrest; both drugs have been successfully combined with metronomic chemotherapy [4,53]. In addition, PPARγ agonists can boost the anti-angiogenic activity of metronomic chemotherapy by increasing endothelial cell expression of CD36, which binds TSP1 and initiates the extrinsic pathway of apoptosis [54]. PPARγ agonists could thus be useful for potentiation of VEGFR2-independent anti-angiogenesis in combination with metronomic chemotherapy. Low-dose metronomic doxorubicin also activates VEGF receptor-independent, endothelial cell extrinsic apoptosis by increasing Fas expression and synergizing with an anti-angiogenic peptide fragment of TSP1 that up regulates FasL [55]. Thus, a variety of non-VEGF receptor-targeting agents offer viable therapeutic options for combination with metronomic chemotherapy-induced anti-tumor innate immunity.

In a prior study, sorafenib increased tumor metastasis when administered at a dose 6 times higher than that used in the present study (150 mg/kg/day vs. 25 mg/kg/day in this study) [56]. At that high dose sorafenib is likely to inhibit VEGFR2 signaling and thereby suppress global immune surveillance, leading to the observed increase in metastasis. Further investigation is required to determine whether a low-dose sorafenib regimen, such as that used here, might effect sufficient anti-angiogenesis while avoiding the increase in metastasis seen with several VEGF pathway inhibitory anti-angiogenic drugs [56,57], and whether it might be effective in combating metastases when combined with intermittent metronomic CPA. It will also be important to determine whether intermittent metronomic chemotherapy can activate anti-tumor innate immunity at metastatic nodules.

While our results establish that the VEGFR2-specific inhibitor DC101 suppresses tumor regression and the recruitment of innate immune cells (Figure 2), our findings do not require that VEGFR2 necessarily be expressed on the tumor-infiltrating innate immune cells themselves, or if it is, that DC101 inhibition of innate immune cell VEGFR2 signaling be the underlying mechanism for the block in immune cell recruitment. Other mechanisms that should be considered include a requirement for VEGFR2 signaling carried out by immune progenitors, or by one or more tumor-associated cells, including stromal and endothelial cells, or perhaps the tumor cells themselves, for attraction of the innate immune cells to the drug-treated tumors. DC101 can suppress endothelial cell progenitor mobilization from the bone marrow in response to chemotherapy [41], suggesting that DC101, and the other VEGF receptor-selective drugs [10], may block tumor recruitment of innate immune cells by inhibiting VEGFR2 signaling required for mobilization of immune cell hematopoietic progenitors [23]. DC101 can also increase local tumor invasiveness and distant liver and lymph node metastasis [57]. Our finding of increased SDF1α expression (Additional file 5), which also occurs during chemotherapy-induced circulating endothelial progenitor mobilization [41], might be indicative of such immune mobilization.

Side effects of VEGF/VEGFR2 antagonists, such as internal bleeding or problems with post-operative wound healing reported for the anti-VEGF monoclonal antibody bevacizumab (Avastin) [58], could result from decreased leukocyte levels, in particular platelets. Bevacizumab binds and inhibits human VEGF, but not mouse VEGF [59], and consequently, systemic side effects such as immune suppression, while observable in human patients, would not be manifested with bevacizumab in preclinical mouse models. Several successful clinical protocols combine VEGF pathway-targeted anti-angiogenic dugs with metronomic CPA (e.g., [60]), however, those protocols commonly use daily low dose CPA delivery, which based on our recent findings would not be expected to activate an innate immune cell response [9].

## Conclusions

The inhibition of anti-tumor innate immunity by VEGF-directed anti-angiogenic drugs involves VEGFR2 as a key target, and likely reflects the expression of VEGFR2 on both immune cell and endothelial cell lineages. The inhibition of innate immune cell recruitment is not due to anti-angiogenesis *per se*, i.e., does not result from the loss of blood vessels trafficking immune cells into the tumor compartment. Importantly, this inhibition can be avoided by using anti-angiogenesis drugs that exert little or no inhibitory activity toward VEGFR2 *in vivo*. Further investigations of the VEGFR2-dependent mechanisms that underlie this inhibition may help elucidate the role of VEGFR2 in other immune responses and in some of the undesirable effects of VEGF pathway-targeted anti-angiogenic drugs.

## Methods

### Cell lines and reagents

The rat 9L gliosarcoma cell line, authenticated by and obtained from the UCSF Neurosurgery Tissue Bank (San Francisco, CA), was grown at 37°C in a humidified, 5% CO_2_ atmosphere in 10% FBS, 100 units/ml penicillin, and 100 μg/ml streptomycin containing DMEM culture medium. CPA was purchased from Sigma Chemical Co. (St. Louis, MO), sorafenib was purchased from LC Labs (Woburn, MA), axitinib was a gift from Pfizer (New York, NY), and DC101 was a gift from ImClone Systems (New York, NY). Fetal bovine serum (FBS) and DMEM were purchased from Invitrogen (Frederick, MD).

### qPCR analysis

Isolation of total RNA from frozen tumor tissue, reverse transcription, and qPCR analysis were carried out using primer sets described previously [10] or shown in Additional file 6. Primers were designed using Primer Express (Applied Biosystems, Carlsbad, CA) and evaluated using LaserGene software (DNAStar, Madison, WI) to ensure mouse-specificity. The absence of cross-species amplification was verified by testing each primer set using a panel of RNA samples isolated from rat and mouse liver, human HUVEC cells, rat 9L cells and human U251 cells. Results were analyzed using the comparative C_T_ (ΔΔC_T_) method and are presented as relative RNA level compared to untreated tumors after normalization to the 18S RNA content of each sample.

### Tumor xenograft studies

ICR/Fox Chase immune deficient male *scid* mice were purchased from Taconic Farms (Germantown, NY) at the age of 5 to 6 weeks (24 to 26 g) and were housed in the Boston University Laboratory of Animal Care Facility and treated in accordance with approved protocols and federal guidelines. 9L cells (4 × 10^6^ cells) were injected *s.c.* on each posterior flank in 0.2 ml serum-free DMEM using a 0.5-inch 29-gauge needle and a 0.3 ml insulin syringe. Tumors volumes and body weights were measured at least twice/wk [10] and treatment groups were normalized (each tumor volume set to 100%) once average volumes reached 500 mm^3^. Mice were treated with CPA on a 6-day repeating metronomic schedule at 140 or 160 mg CPA/kg body weight (BW)/injection (equal to doses of ~150 and 170 mg/kg, respectively, based on CPA-monohydrate [9]), which induce indistinguishable immune responses and anti-tumor activity in this model [9]. Sorafenib and axitinib were administered daily at 25 mg/kg BW/day i.p. for up to 36 days. The VEGFR2-specific monoclonal antibody DC101 was administered *i.p.* every 3 days at 22.5 mg/kg BW, or as specified, once the tumors reached an average volume of ~360 mm^3^. On co-treatment days, DC101, or sorafenib, was administered 4 hr after CPA to minimize the potential for drug-drug interactions. Day 0 marks the first day of drug treatment. 9L tumors are well tolerated when grown s.c. in this animal model and show little or no toxicity to the mouse host, even at much larger tumor sizes [37].

### Tissue processing and immunohistochemistry

Tumors were collected on day 0 (first day of drug treatment) and 6 days after the second and fourth cycles of CPA treatment, i.e., days 12 and 24, or as indicated. Tumors were excised and portions were frozen in liquid nitrogen (for RNA) or in 2-methylbutane (for tissue sectioning and immunohistochemistry). Cryosections (5–10 μm) were prepared and fixed in acetone (for NK1.1 and Prf1 immunostaining) or 1% paraformaldehyde (for CD31 staining). Slides were stained using goat anti-mouse NK1.1 (CD161) antibody (clone M-15, cat. #sc-70150, Santa Cruz Biotech, Santa Cruz, CA) at a dilution of 1:25, or rat anti-mouse CD31 (Cat. #557355, BD Biosciences Pharmingen, San Jose, CA) at a dilution of 1:1000, or rat anti-mouse Perforin 1 (CB5.4) antibody (cat. #sc-58643, Santa Cruz Biotech) at a dilution of 1:100. An avidin-biotin blocking kit (cat. #SP-2001, Vector Labs, Burlingame, CA) was used for all incubations. Incubation with biotinylated rabbit anti-goat (cat. #BA-5000; 1:250 dilution) (for NK1.1) or rabbit anti-rat (cat. #BA-4000; 1:200 dilution) (for CD31 and Perforin 1) secondary antibody was followed by ABC signal amplification with Vectastain ABC peroxidase reagent (cat. #PK-4000 or cat. #PK-6100), and then color development with VIP (cat. #SK-4600) or DAB (cat. #SK-4100) substrate (all from Vector Labs). For paraffin-embedded samples (DC101 study), CD31 antibody (Dianova, Hamburg; cat. #DIA 310) was used at a dilution of 1:40 along with ABC Elite (Vector, cat. #PK-6100) for enhancement and ImmPACT VIP for detection (Vector, cat. #SK-4605). The washed slides were briefly countered stained with Hematoxylin Solution, Harris Modified (Sigma, cat. #HHS128). Stained tumor section images were quantified using NIH ImageJ, typically based on ≥10-20 images per group [10]. Data shown are treatment group mean values ± S.E.

### Tumor VEGFR phosphorylation

Mice bearing 9L tumors ~500 mm^3^ in size were treated with a single i.p. injection of DC101 (22.5 mg/kg BW), sorafenib (25 mg/kg BW), or axitinib (25 mg/kg BW). Tumors were collected after 2 hr (sorafenib, axitinib), based on the reported half-lives of ~ 2 hr for those drugs [31,61], or after 4 hr (DC101), to give sufficient time for DC101 absorption and distribution [62]. The effects of these drug treatments on the activity state of functional tumor blood vessel VEGFR was assessed by immunohistochemical staining for VEGFR phosphorylation. VEGFR phosphorylation of tumor blood vessels could not be reliably assayed after multiple days of anti-angiogenic drug treatment owing to the extensive loss of tumor blood vessels (e.g., Figure 2B and Figure 3B). 9L tumor cryosections were fixed with 4% paraformaldehyde for 15 min, permeabilized with 1% Triton X-100 in 1% sodium citrate for 5 min at 4°C, and then blocked with fresh 3% H_2_O_2_ for 5 min at room temperature, followed by 5% normal horse serum for 20 min at room temperature. Slides were incubated with anti-phospho-VEGFR2 antibody (rabbit anti-mouse phospho-Fik-1, Tyr1214, 1:25 dilution; sc-101820, Santa Cruz Biotechnology) overnight at 4°C, followed by biotinylated horse anti-rabbit secondary antibody (BA-1100, Vector Labs; 1:250 dilution) for 60 min at room temperature, then ABC Vectastain Elite for 30 min at room temperature, DAB (SK-4100, Vector labs) for 10 min for detection, followed by hematoxylin counterstaining.

### FACS analysis

Single-cell suspensions of freshly excised spleens and tumors were prepared in a GentleMACS Dissociator (Miltenyi Biotec, Auburn, CA) using the manufacturer’s protocols for mouse spleens and implanted tumors. Bone marrow was isolated from excised mouse femurs and tibiae, which were cut open at the ends with surgical razor blades and washed out with phenol red-free alpha-MEM (Cat. #41061, Gibco, Grand Island, NY). Tumor single-cell suspensions (5 ml) were diluted to 15 ml with PEB dissociation buffer (1X PBS, pH 7.2, 0.5% BSA, and 2 mM EDTA). Diluted tumor, bone marrow, and spleen suspensions were passed through 70 μm filters (Cat. #22363548, Fisher Scientific, Pittsburgh, PA). All three tissue-derived, single-cell populations were subjected to red blood cell lysis with 5 ml of 1X RBC lysis buffer (Cat. #00-4333, eBioscience, San Diego, CA) for 5 min at room temperature. Reactions were terminated by addition of 20 ml of sterile 1X PBS. Cells remaining were centrifuged at 300-400 g at 4°C and resuspended in ~50 μl of eBioscience Staining Buffer for antibody incubation. Spleen samples were incubated with 0.5-1 μg of anti-CD16/CD32 antibody (Cat. #14-0161-82, eBioscience) for 5–10 min on ice to neutralize non-specific Fc antibody interactions. All samples were incubated in the dark for 25 min at 4°C with fluorescently tagged monoclonal antibody specific for the cell markers Ly-6G (Gr-1) (1 μl (0.5 μg) per sample; Ly-6G-PE, Clone RB6-8C5, Cat. #11-5931, eBioscience) and CD11b (1 μl (0.2 μg) per sample; CD11b-PE, Clone M1/70, Cat. #12-0112, eBioscience). Background samples were stained with FITC- and PE-labeled Rat IgG (1 μl per sample, Cat. #553929 and Cat. #12-4321, eBioscience). Samples were washed, filtered, resuspended and analyzed [10].

### Statistical analysis

Data were analyzed for statistical significance by one-way ANOVA with Bonferroni multiple comparison correction (for comparisons involving 3 or more groups) or by t-test, two-tailed, unpaired comparison, as implemented in GraphPad Prism 4.1.

## Abbreviations

BW: Body weight; CPA: Cyclophosphamide; DC101: VEGFR2-specific inhibitory monoclonal antibody; NK: Natural killer; MDSC: Myeloid-derived suppressor cell; RTKI: Receptor tyrosine kinase inhibitor; TSP1: Thrombospondin-1; VEGFR2: VEGF receptor 2.

## Competing interests

The authors declare that they have no competing interests.

## Authors’ contributions

JCD contributed to concept design, carried out animal studies and molecular analyses, and drafted the manuscript jointly with DJW. CS carried out additional animal studies and molecular characterization. DJW helped conceive the study, coordinated the overall project and revised and edited the manuscript. All authors read and approved the final manuscript.

## Supplementary Material

Additional file 1 **FACS analysis of metronomic CPA-treated 9L tumors removed 6 days after the 2**^**nd **^**and 4**^**th **^**cycles of CPA treatment (days 12 and 24, respectively), compared to untreated (UT) 9L tumors collected on day 24.** Cell numbers in each quadrant are expressed as a percentage of the total cell population. Data for untreated tumors and for day 24 tumors is the same as presented in Figure 1 and is included here for direct comparison. Data show a large time-dependent increase in CD11b single-positive cells with metronomic CPA treatment. Click here for file

Additional file 2 **qPCR data supporting DC101 inhibition of anti-tumor innate immune function in metronomic CPA co-treated tumors.** Analysis of host (m, mouse) NK cell markers NKG2D, macrophage lymphocyte marker Fas and Fas ligand (FasL) in 9L tumor xenografts that were treated as in Figure 2 and isolated from untreated (UT) tumors (day 15), and at a time point corresponding to 4 CPA treatment cycles (DC101 day 21, CPA day 24, CPA + DC101 day 24). Tissue RNA samples analyzed are the same ones shown in Figure 2C. Bars, mean ± SE for n=10-12 tumors/group. **, ***, *p*< 0.01 or 0.001, treatment versus UT; ^^, ^^^, *p*< 0.01 or 0.001, for CPA + DC101 versus CPA alone, by 1-way ANOVA with Bonferroni multiple comparison correction, implemented in GraphPad Prism 4. Click here for file

Additional file 3 **DC101 inhibits metronomic CPA-induced innate immune recruitment.** Immunohistochemical staining of the macrophage marker CD68, the NK cell marker NK1.1, and perforin 1 in untreated 9L tumors (tumors excised on treatment day 15; see Figure 2A) and in 9L tumors treated with DC101 alone and collected on treatment day 21, or treated metronomic CPA with or without DC101, and collected on treatment day 24. Magnification is as shown at the bottom. Click here for file

Additional file 4 **Effect of anti-angiogenic drugs on 9L tumor VEGFR phosphorylation.** Cryosections prepared from tumors treated with the indicated anti-angiogenic drugs were stained with antibody to VEGFR2 phosphotyrosine-1214 and counterstained with hematoxylin as described under Methods. Shown are representative images of each treatment group. Partial inhibition was apparent in some of the sorafenib-treated sections, and inhibition was incomplete at the lower dose of DC101 (10 mg/kg). Blue arrows mark phospho-VEGR2-stained blood vessels. Click here for file

Additional file 5 **Impact of metronomic CPA treatment in combination with sorafenib on additional innate immune cell markers.** qPCR analysis of host (m, mouse) dendritic cell marker CD74 (A), interferon-producing natural killer cell (IKDC/NKDC) marker B220 (B), platelet-associated marker platelet factor 4 (Pf4) (C), platelet-associated factor stromal-derived factor 1-alpha (SDF1α) (D), and bone marrow-derived cell (i.e., monocyte) marker CD11b (E) in 9L tumors grown in *scid* mice, treated with vehicle, sorafenib alone, or metronomic CPA ± sorafenib and isolated at various time points throughout treatment (6 days after the 2^nd^, 4^th^, and 6^th^ CPA cycles: days 12, 24, and 36). Samples analyzed are the same as shown in Figure 4 and Figure 5. Bars, mean ± SE for n = 5–6 tumors/group. Click here for file

Additional file 6 **Mouse-specific (host) forward and reverse primer sets used for qPCR analysis of RNA levels.** Primers were designed to anneal at their 3’ end in a mouse (host)-specific manner. Species alignments between human, rat, and mouse sequences were used for each gene to determine primer set specificity. The absence of cross-species amplification was verified by testing primer sets on a panel of rat, mouse, and human RNAs to ensure species-specificity, as described above. Official gene names are shown in parentheses. Primer sets for platelet factor 4 (Cxcl4, *Pf4*), Fas receptor (*Fas*), NKp46 (Ncr1), perforin (*Prf1*), granzyme B (*GzmB*), Natural Killer Cell Factor 1.1 (NK1.1, *Klrb1c*), NK chemoattractant CXCL14 (*Cxcl14*), NK cell receptor NKG2D (*Klrk1*), macrophage markers CD68 (*Cd68*) and F4/80 (*Emr1*), macrophage cytolytic effectors lysozymes 1 and 2 (*Lyz1* and *Lyz2*), dendritic cell marker CD74 (*Cd74*), neutrophil marker Gr1 (*Ly6g*), endothelial cell marker CD31 (*Pecam1*), and Thrombospondin-1 (TSP1, *Thbs1*) are the same as described [10]. Click here for file
