# Supplementary figures and images for: Anti-tumor innate immunity activated by intermittent metronomic cyclophosphamide treatment of 9L brain tumor xenografts is preserved by anti-angiogenic drugs that spare VEGF receptor 2
Source: Mol Cancer. 2014 Jun 26;13:158. doi: 10.1186/1476-4598-13-158 (PMC4083145; doi:10.1186/1476-4598-13-158)

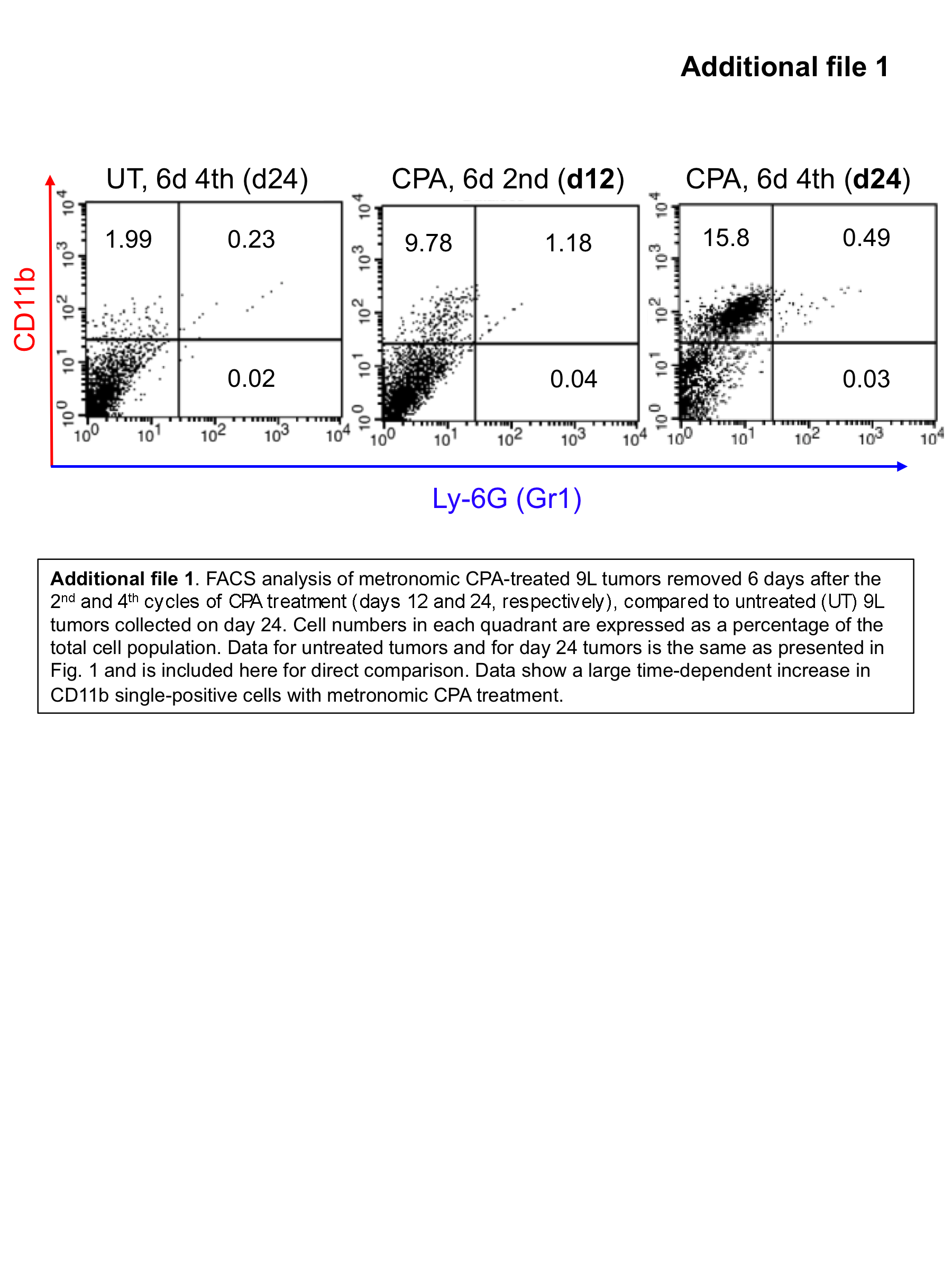

Supplement: Additional file 1 — FACS analysis of metronomic CPA-treated 9L tumors removed 6 days after the 2 nd and 4 th cycles of CPA treatment (days 12 and 24, respectively), compared to untreated (UT) 9L tumors collected on day 24. Cell numbers in each quadrant are expressed as a percentage of the total cell population. Data for untreated tumors and for day 24 tumors is the same as presented in Figure 1 and is included here for direct comparison. Data show a large time-dependent increase in CD11b single-positive cells with metronomic CPA treatment. [file 1476-4598-13-158-S1.png]

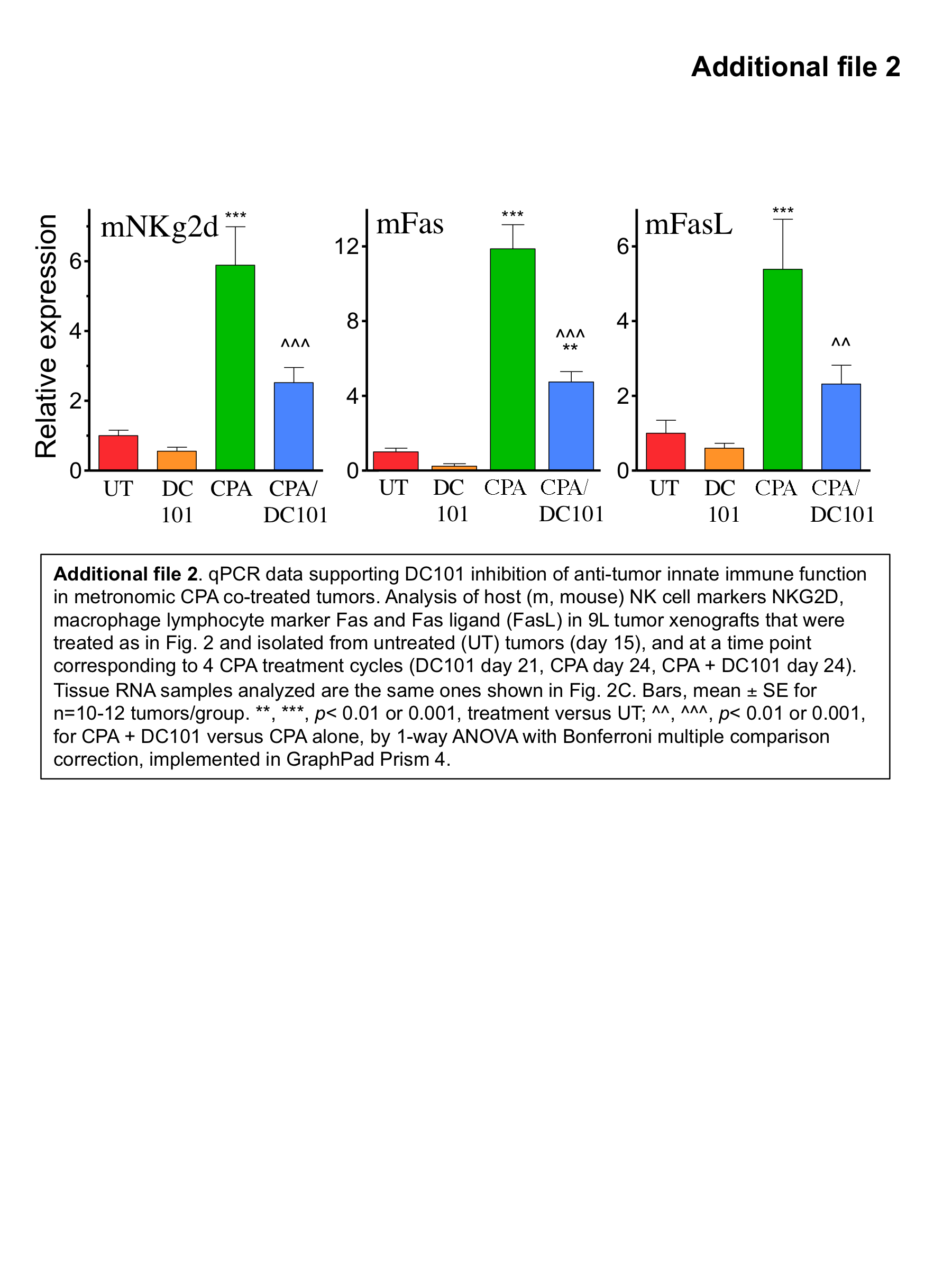

Supplement: Additional file 2 — qPCR data supporting DC101 inhibition of anti-tumor innate immune function in metronomic CPA co-treated tumors. Analysis of host (m, mouse) NK cell markers NKG2D, macrophage lymphocyte marker Fas and Fas ligand (FasL) in 9L tumor xenografts that were treated as in Figure 2 and isolated from untreated (UT) tumors (day 15), and at a time point corresponding to 4 CPA treatment cycles (DC101 day 21, CPA day 24, CPA + DC101 day 24). Tissue RNA samples analyzed are the same ones shown in Figure 2C. Bars, mean ± SE for n=10-12 tumors/group. **, ***, p< 0.01 or 0.001, treatment versus UT; ^^, ^^^, p< 0.01 or 0.001, for CPA + DC101 versus CPA alone, by 1-way ANOVA with Bonferroni multiple comparison correction, implemented in GraphPad Prism 4. [file 1476-4598-13-158-S2.png]

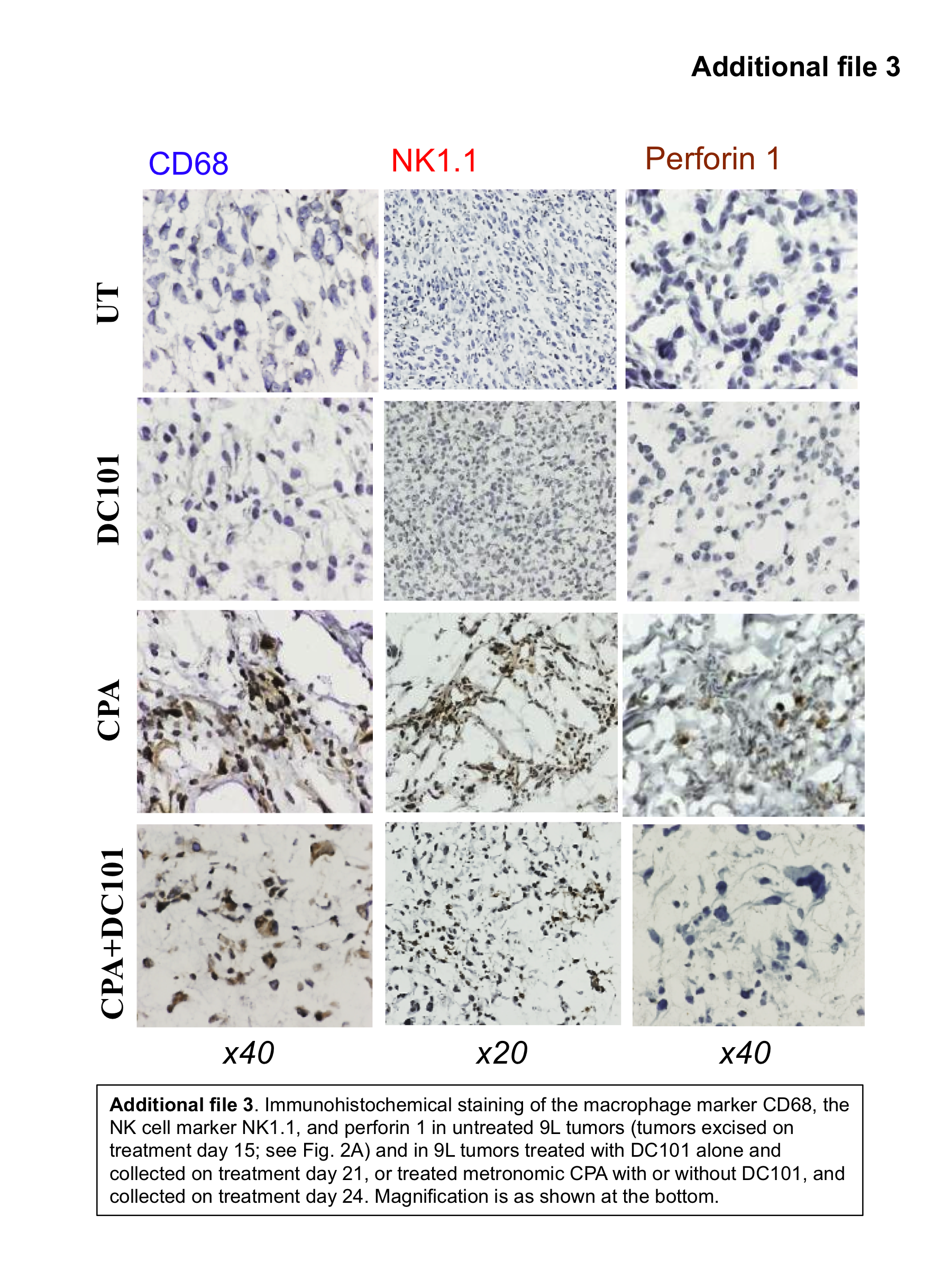

Supplement: Additional file 3 — DC101 inhibits metronomic CPA-induced innate immune recruitment. Immunohistochemical staining of the macrophage marker CD68, the NK cell marker NK1.1, and perforin 1 in untreated 9L tumors (tumors excised on treatment day 15; see Figure 2A) and in 9L tumors treated with DC101 alone and collected on treatment day 21, or treated metronomic CPA with or without DC101, and collected on treatment day 24. Magnification is as shown at the bottom. [file 1476-4598-13-158-S3.png]

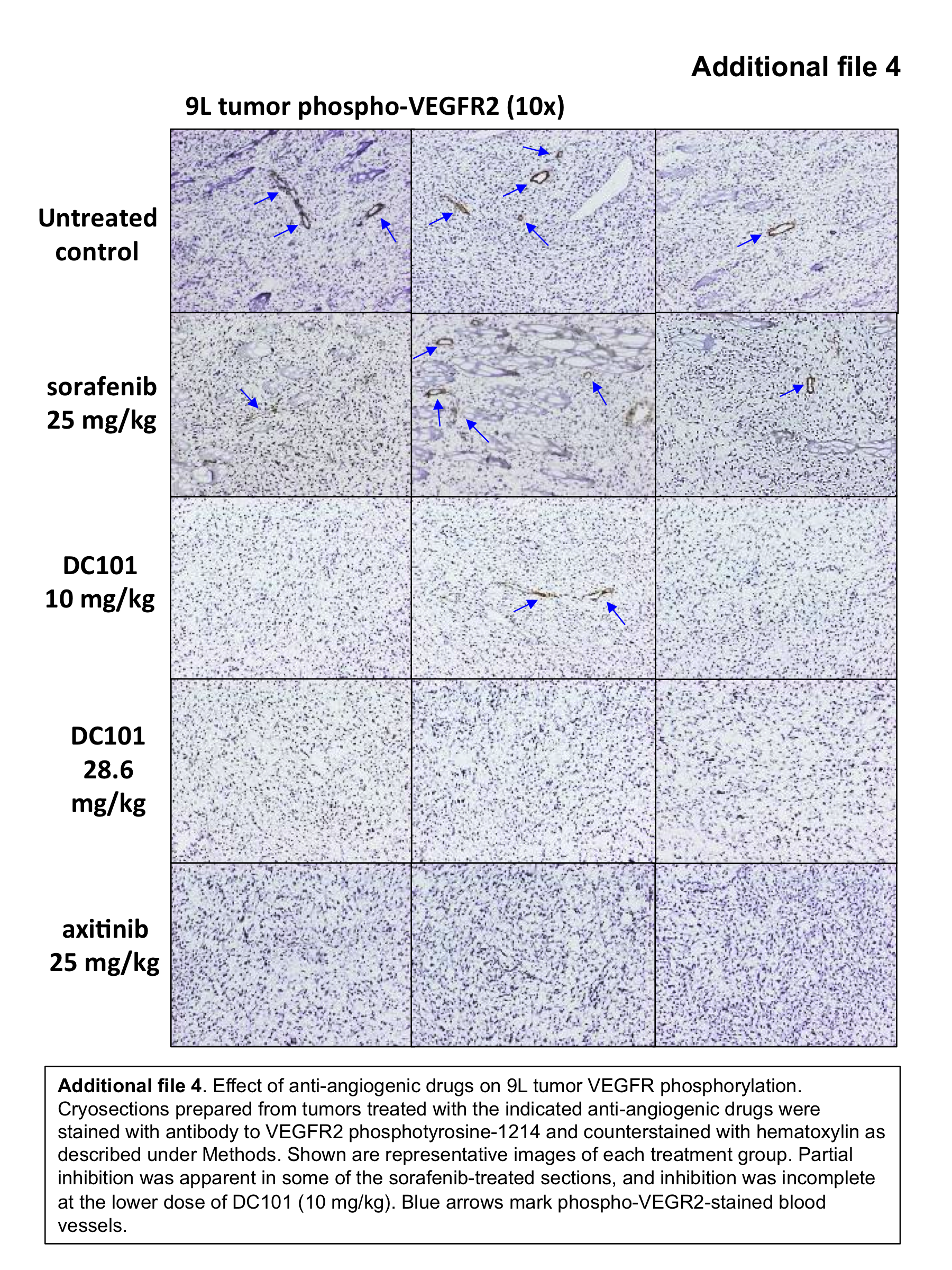

Supplement: Additional file 4 — Effect of anti-angiogenic drugs on 9L tumor VEGFR phosphorylation. Cryosections prepared from tumors treated with the indicated anti-angiogenic drugs were stained with antibody to VEGFR2 phosphotyrosine-1214 and counterstained with hematoxylin as described under Methods. Shown are representative images of each treatment group. Partial inhibition was apparent in some of the sorafenib-treated sections, and inhibition was incomplete at the lower dose of DC101 (10 mg/kg). Blue arrows mark phospho-VEGR2-stained blood vessels. [file 1476-4598-13-158-S4.png]

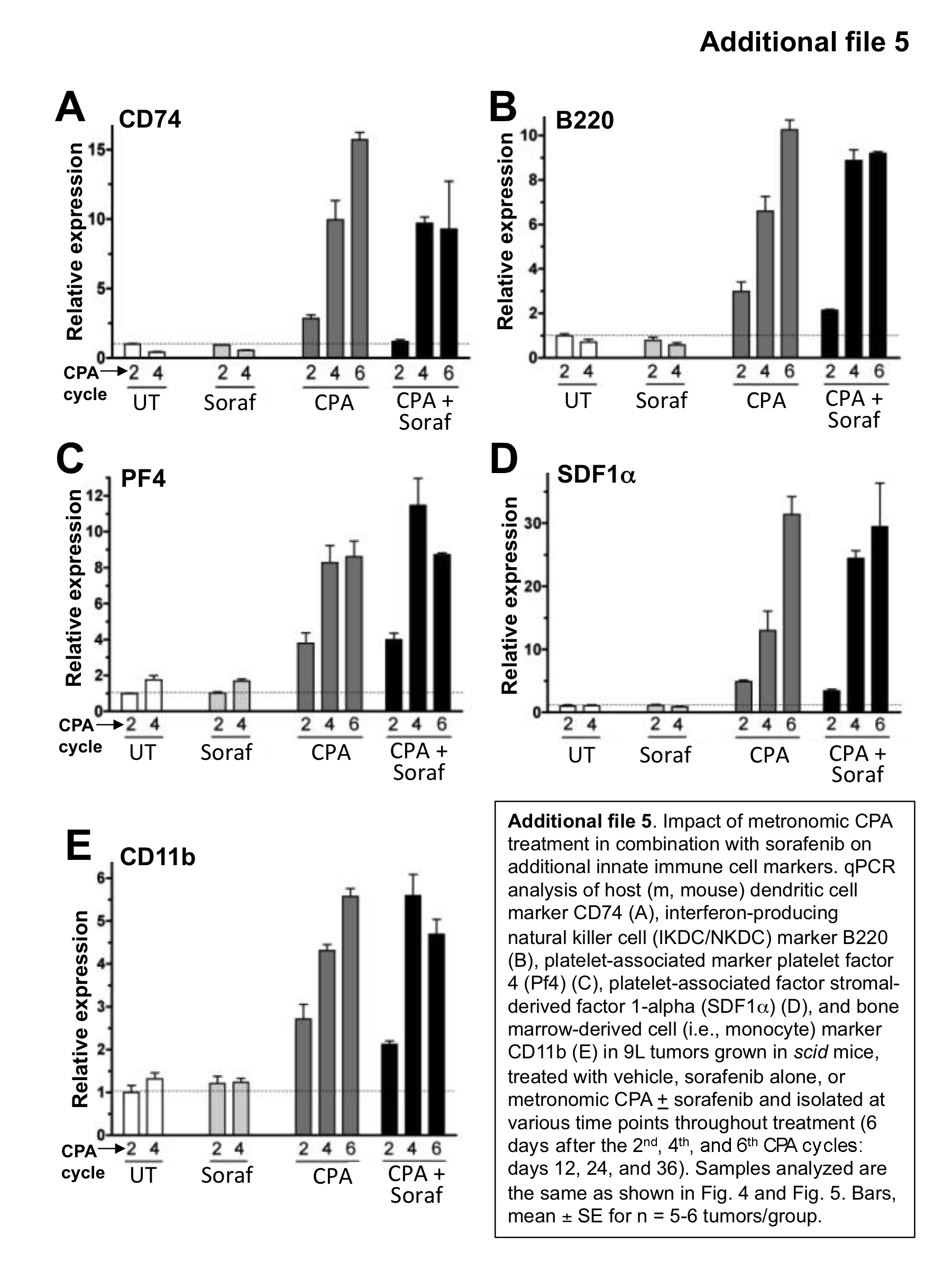

Supplement: Additional file 5 — Impact of metronomic CPA treatment in combination with sorafenib on additional innate immune cell markers. qPCR analysis of host (m, mouse) dendritic cell marker CD74 (A), interferon-producing natural killer cell (IKDC/NKDC) marker B220 (B), platelet-associated marker platelet factor 4 (Pf4) (C), platelet-associated factor stromal-derived factor 1-alpha (SDF1α) (D), and bone marrow-derived cell (i.e., monocyte) marker CD11b (E) in 9L tumors grown in scid mice, treated with vehicle, sorafenib alone, or metronomic CPA ± sorafenib and isolated at various time points throughout treatment (6 days after the 2nd, 4th, and 6th CPA cycles: days 12, 24, and 36). Samples analyzed are the same as shown in Figure 4 and Figure 5. Bars, mean ± SE for n = 5–6 tumors/group. [file 1476-4598-13-158-S5.png]

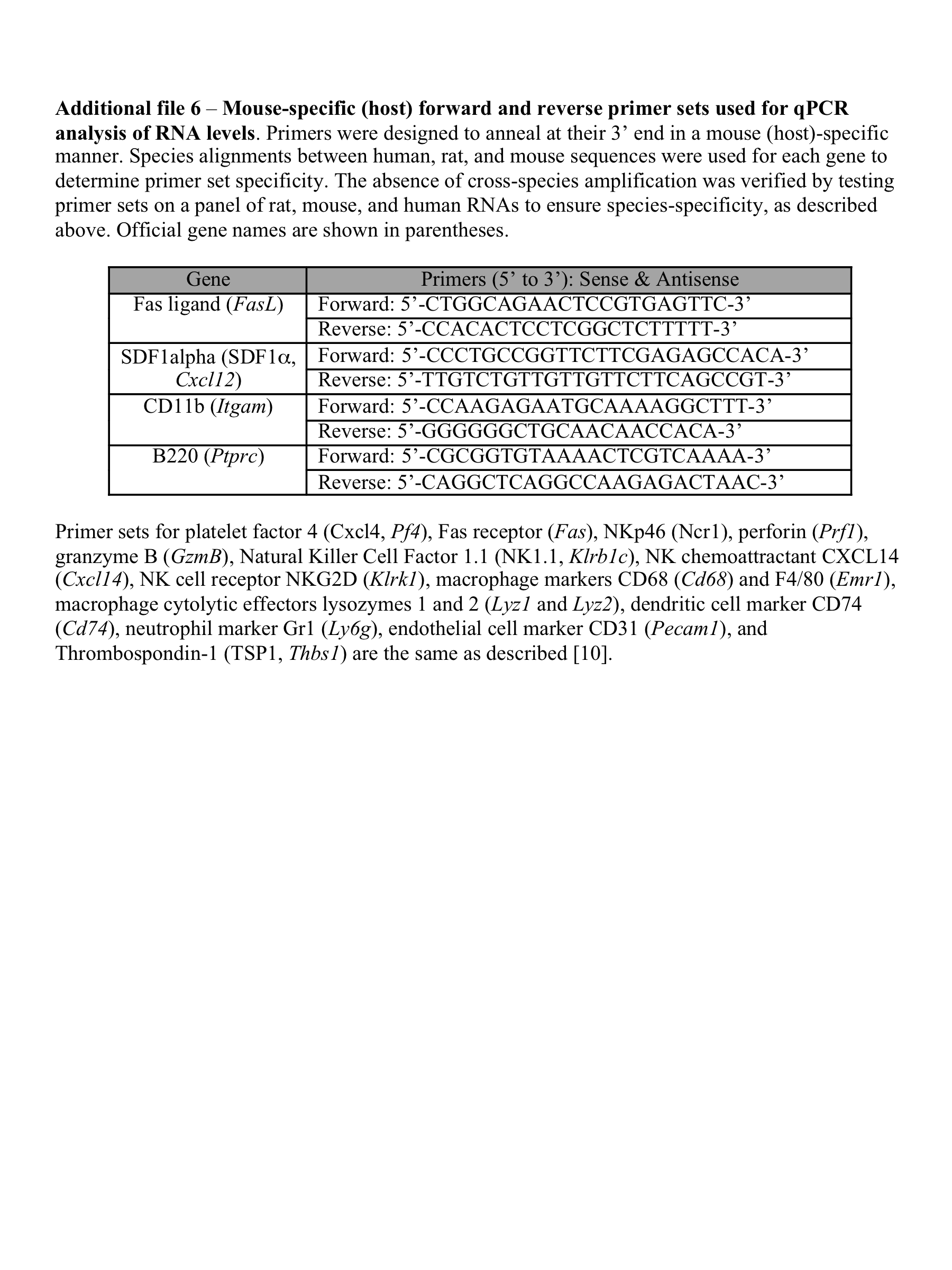

Supplement: Additional file 6 — Mouse-specific (host) forward and reverse primer sets used for qPCR analysis of RNA levels. Primers were designed to anneal at their 3’ end in a mouse (host)-specific manner. Species alignments between human, rat, and mouse sequences were used for each gene to determine primer set specificity. The absence of cross-species amplification was verified by testing primer sets on a panel of rat, mouse, and human RNAs to ensure species-specificity, as described above. Official gene names are shown in parentheses. Primer sets for platelet factor 4 (Cxcl4, Pf4), Fas receptor (Fas), NKp46 (Ncr1), perforin (Prf1), granzyme B (GzmB), Natural Killer Cell Factor 1.1 (NK1.1, Klrb1c), NK chemoattractant CXCL14 (Cxcl14), NK cell receptor NKG2D (Klrk1), macrophage markers CD68 (Cd68) and F4/80 (Emr1), macrophage cytolytic effectors lysozymes 1 and 2 (Lyz1 and Lyz2), dendritic cell marker CD74 (Cd74), neutrophil marker Gr1 (Ly6g), endothelial cell marker CD31 (Pecam1), and Thrombospondin-1 (TSP1, Thbs1) are the same as described [10]. [file 1476-4598-13-158-S6.png]
